# Belowground interactions shift the relative importance of direct and indirect genetic effects

**DOI:** 10.1002/ece3.582

**Published:** 2013-05-02

**Authors:** Mark A Genung, Joseph K Bailey, Jennifer A Schweitzer

**Affiliations:** Department of Ecology and Evolutionary Biology, University of Tennessee – Knoxville569 Dabney Hall, Knoxville, Tennessee, 37996

**Keywords:** Aboveground, belowground, community and ecosystem genetics, evolution, genetic variation, indirect genetic effects, plant-neighbor interactions, *Solidago*

## Abstract

Intraspecific genetic variation can affect decomposition, nutrient cycling, and interactions between plants and their associated belowground communities. However, the effects of genetic variation on ecosystems can also be indirect, meaning that genes in a focal plant may affect ecosystems by altering the phenotype of interacting (i.e., neighboring) individuals. We manipulated genotype identity, species identity, and the possibility of belowground interactions between neighboring *Solidago* plants. We hypothesized that, because our plants were nitrogen (N) limited, the most important interactions between focal and neighbor plants would occur belowground. More specifically, we hypothesized that the genotypic identity of a plant's neighbor would have a larger effect on belowground biomass than on aboveground biomass, but only when neighboring plants were allowed to interact belowground. We detected species- and genotype-level variation for aboveground biomass and ramet production. We also found that belowground biomass and ramet production depended on the interaction of neighbor genotype identity and the presence or absence of belowground interactions. Additionally, we found that interspecific indirect genetic effects (IIGEs; changes in focal plant traits due to the genotype identity of a heterospecific neighbor) had a greater effect size on belowground biomass than did focal genotype; however, this effect only held in pots that allowed belowground interactions. These results expand the types of natural processes that can be attributed to genotypes by showing that, under certain conditions, a plant's phenotype can be strongly determined by the expression of genes in its neighbor. By showing that IIGEs are dependent upon plants being able to interact belowground, our results also provide a first step for thinking about how genotype-based, belowground interactions influence the evolutionary outcomes of plant-neighbor interactions.

## Introduction

While it is becoming established that intraspecific genetic variation can influence associated communities and ecosystems (e.g., Johnson and Agrawal 2005; Bailey et al. 2006; Crutsinger et al. 2006; Johnson et al. 2006; Whitham et al. 2006; Fridley et al. 2007; Mooney and Agrawal 2008), how genetically-based species interactions influence belowground plant traits that are of critical importance to plant competition, nutrient cycling and overall plant fitness is poorly understood. Understanding the aboveground effects of intraspecific genetic variation is important because of its effects on associated communities (Crutsinger et al. 2006; Whitham et al. 2006; Genung et al. 2012), plant fitness (e.g., Johnson et al. 2006), species interactions (Bailey et al. 2006; Mooney and Agrawal 2008), and many other ecological patterns and processes. However, genetic variation can also drive belowground interactions that affect plant fitness and nutrient cycling (e.g., Madritch et al. 2006; Schweitzer et al. 2004; Pregitzer et al. 2010), as well as the belowground communities associated with plant roots, such as soil arthropods and microorganisms (Schweitzer et al. 2008; Crutsinger et al. 2009). Compared to research at the species level, research into belowground plant-neighbor interactions at the genotype level has received less attention. For example, most plant-neighbor studies have looked at the physiological mechanisms of resource competition or the population and community impacts of species-level competition (see Casper and Jackson 1997 for review). Additionally, the relatively few community and ecosystem genetics studies that have looked at belowground plant traits (e.g., Bossdorf et al. 2009; Collins et al. 2010; Genung et al. 2012) have measured total belowground biomass, which is sometimes a poorer predictor of nutrient uptake than other metrics such as root surface area (Caldwell et al. 1991).

Genotype-level studies of belowground plant-neighbor interactions have additional implications, as there are immediate evolutionary consequences if neighbor genotype effects are interpreted as indirect genetic effects (IGEs). IGEs are environmental influences on the phenotype of a focal species due to the expression of genes in an interacting, conspecific individual (Moore et al. 1997). IGEs can also occur between members of different species, and when this occurs they are termed interspecific indirect genetic effects (IIGEs; Shuster et al. 2006). As opposed to IGEs, which influence social evolution, IIGEs affect species interactions and community change. IIGEs are contingent on a significant effect of “neighbor genotype” on phenotypic traits in a focal plant. If the IIGE is mediated by belowground interactions between a focal plant and its neighbors, and the affected focal plant trait is heritable and has consequences for plant fitness, then belowground interactions may affect genotype frequencies in the next generation by altering the performance and survival of particular genotypes. Understanding the relative roles of direct (genotype) versus indirect (neighbor genotype) genetic effects on plant phenotypes, and determining whether the importance of these factors varies across plant traits (i.e., aboveground biomass, belowground biomass, ramet production, root surface area) or environments, represents an important step for understanding how IIGEs affect belowground interactions.

The importance of understanding how genotypic variation and IIGEs affect the outcome of belowground interactions between neighboring plants is underscored by the observation that plant performance is affected more by belowground competition than by aboveground competition (Wilson 1988). There exists a rich history of belowground competition studies, both at the physiological level and at the population/community level (Casper and Jackson 1997 and references therein). However, to our knowledge, these studies have not taken the perspective of comparing the relative roles of genotypic effects and IIGEs to understand more about how evolution and coevolution may occur in response to belowground interactions. For example, IIGEs may have strong effects when they originate in abundant species with major impacts on ecosystem function (i.e., foundation species), and weaker effects when they originate in rare species. Another possibility is that IIGEs are strongest for traits related to acquiring limiting nutrients (Genung et al. 2012), because interactions involving these traits have presumably been of significant evolutionary importance. Comparing the effect size of genotypic variation with other ecological and evolutionary factors such as belowground interactions and IIGEs will help inform a broader effort (e.g., Bailey et al. 2009) to understand the relative importance of genotypic variation for associated community structure and ecosystem processes.

**Figure 1:**
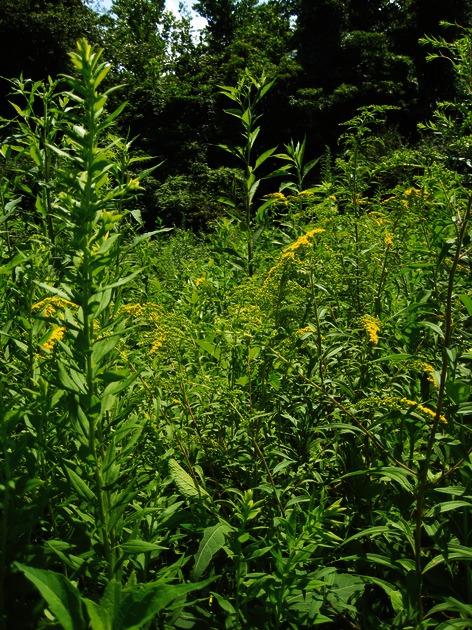
*Solidago altissima* (left) and *Solidago gigantea* (right) co-occurring in an old field in Knox County, TN.

Using three genotypes each of *Solidago altissima* and *Solidago gigantea* (Fig. 1), we established a common garden experiment that manipulated genotype identity, neighbor genotype identity, and the possibility of belowground interactions to examine the effects of interspecific genotype interactions on aboveground plant biomass, belowground plant biomass, ramet production, and root surface area. The possibility of interactions was determined by planting paired plants in custom-made planting boxes that either allowed interactions (no barrier, i.e., “undivided pots”) or prevented belowground interactions (water-tight barrier between individual plants, i.e., “divided pots”). This experiment allows us to examine how intraspecific genetic variation (i.e., “focal genotype”) and biotic environmental variation (i.e., “neighbor genotype” or IIGEs) interact to affect the outcome of plant-neighbor interactions. Given that *Solidago* is generally N limited and because, using these same genotypes, we observed increased biomass following N fertilization in a previous study (Genung et al. 2012), we hypothesized that the effect size of neighbor genotype would be largest in pots where belowground interactions were allowed to occur, and that neighbor genotype would have little to no effect on focal plants in the absence of belowground interactions. However, neighboring plants could potentially compete for light, affect each other's susceptibility to herbivores, or release aboveground volatile chemicals, suggesting that some plant-plant interactions may still occur in divided pots. Our main question asked whether excluding belowground interactions altered the effects of focal genotype and neighbor genotype; specifically, we were interested in interactive effects between the pot division treatment and either focal or neighbor genotype. We tested this question in two-species mixtures, where focal and neighbor genotype were different, and in genotype monocultures. Secondarily, we tested whether root surface area or belowground biomass was a better predictor of aboveground biomass. We found that species- and genotype-level variation affected aboveground biomass and ramet production, and that neighbor genotype identity interacted with the pot division treatment to affect belowground biomass and ramet production. These results support the idea IIGEs (i.e., neighbor genotype effects) have different effects on host plants when belowground interactions are experimentally excluded.

## Methods

### Study species

*Solidago altissima* is a dominant species in abandoned agricultural fields where it can have large impacts on biodiversity and ecosystem function (Maddox and Root 1987; Crutsinger et al. 2006). *S. altissima* frequently co-occurs with *S. gigantea* in old fields (Abrahamson et al. 2005), although the two species differ in a range of life-history traits (Abrahamson and Weis 1997; Abrahamson et al. 2005; Genung et al. 2012). *S. altissima* is highly clonal and produces more rhizome biomass than *S. gigantea*, while *S. gigantea* allocates a greater percentage of its biomass to inflorescences (Abrahamson et al. 2005). *S. altissima* and *S. gigantea* are both known to produce shorter rhizomes, and overall less rhizome biomass, in fertilized soil relative to unfertilized soil (Schmid and Bazzaz 1992), suggesting that belowground biomass in these species is plastic with regard to soil nutrient availability. Intraspecific genetic variation in *S. altissima* has been shown to affect ecosystem level responses (e.g., Crutsinger et al. 2006, 2009; Genung et al. 2012). Previous work with the genotypes used in this experiment has shown that the *S. altissima* genotypes used in this study vary in rhizome biomass, while the *S. gigantea* genotypes differ in coarse root biomass, aboveground vegetative biomass, and floral biomass (Genung et al. 2012).

### Garden design

In March 2010, a common garden experiment was established at the East Tennessee Research and Education Center in Knoxville, Tennessee. This common garden included three locally collected genotypes (i.e., clonal families) of both *S. altissima* and *S. gigantea*. The *S. altissma* and *S. gigantea* clones we utilized were originally propagated by G.M. Crutsinger and clones were maintained at the University of Tennessee. The genotypes were collected from random locations around the study site at Freels Bend; sampled individuals from both species were carefully collected from unique connected genets that were at least 50–150 m apart (Crutsinger et al. 2006, Supplementary Material) and these were assumed to be genetically distinct. Rhizomes were collected from connected ramets to ensure they were from the same genet. The three *S. altissima* genotypes were originally determined as unique genotypes using amplified fragment length polymorphism data (Crutsinger et al. 2006, Supplementary Material); however, molecular data is unavailable for the *S. gigantea* genotypes. Because only three genotypes were used, we stress that we are not attempting to represent the full range of variation expressed in our species, but rather intend our experiment to be a proof-of-concept for what is possible when genotypes of different species interact in natural systems.

The experimental treatments included genotype monocultures as well as all possible interspecific combinations of *S. altissima* and *S. gigantea* genotypes, planted together in custom built, open-top cubic containers (each side = 0.33 m). Because we chose to focus on interspecific combinations, no intraspecific genotype combinations were included in this study. Half of the containers were centrally divided using a waterproof, airproof, polypropylene sheet to create two equal halves, a design that aimed to prevent belowground interactions from occurring in these containers. Although this treatment could potentially reduce the amount of area a plant in the divided treatment could explore relative to a strong competitor in the undivided pots (i.e., we kept total pot-level resources constant, meaning that accessible resources varied in divided and nondivided pots), we rarely observed root-bound plants when belowground biomass was collected, and we found no differences in total plant biomass in divided pots versus open pots. Treatments consisted of interspecific genotype-neighbor genotype pairs (i.e., *S. altissima* genotype A1 grown with *S. gigantea* genotype G1) either in divided pots or undivided pots. There were six genotypes monoculture (one for each genotype), nine genotypes mixtures (all factorial combinations of 3 *S. altissima* genotypes × 3 *S. gigantea* genotypes), and presence/absence of belowground interactions (excluded or permitted) for a total of 30 treatments. We replicated each treatment seven times for a total of 210 pots, or 420 plants. Due to certain analyses focusing on monoculture pots versus two-species pots, and because of the subsampling of belowground biomass (described later), sample size is sometimes lower; the number of samples used in each analysis is given in the tables.

All plants were propagated from cloned stocks of genotypes. A 3-cm rhizome of each species and genotype were grown, in greenhouse flats, outdoors in shaded conditions, and watered as needed. When the plants were *c*. 15 cm in height they were transplanted into the pots at the field site. After transplanting, the initial aboveground biomass of individuals was estimated using an allometric equation (Weight (g) = (−0.071 + 0.0346 × height (cm)^2^; *r*^2^ = 0.83). We initially used initial biomass as a covariate in our analyses, but this did not affect our results so we excluded initial biomass to prevent biasing against direct genetic effects (genotype effects) that occurred before transplanting. Each pot initially included two individuals, but variation in plant density occurred due to clonal production of new ramets beginning during the growing season (2010). In monocultures, both individuals were clones of the same genotype. In genotype mixtures, each pot initially contained one individual of each genotype (two plants total/pot). The pots were randomly placed in a grid formation within an old field with *c*. 1 m separating each pot from its neighbors. The surrounding field was mown frequently during the experiment, and supplementary water was added to each pot in equal amounts when conditions required. Water was allowed to drain through small holes drilled into the bottom of the pots. The bottom quarter of the pots was filled with gravel (to aid draining). Inside the pots, the gravel was covered with shade cloth and Sunshine Growing Mix #4 (Sun Gro Horticulture, Vancouver, British Columbia, Canada). Invading plants were removed throughout the experiment. Approximately 10 g of fertilizer (24/8/16, Miracle-Gro, Marysville, OH) was applied once to each pot in April 2010.

### Trait measurements

After 9 months of growth, we measured belowground plant biomass at the conclusion of the growing season by destructively sampling a subset of 100 pots. A subset of pots was used because our methods for determining belowground biomass and root surface area were labor-intensive. We removed entire blocks of soil from the pots, and water-filtered soil through a 1 mm sieve (USA Standard Testing #18) to remove all roots (i.e., rhizomes and both coarse and fine roots) from the soil. In all divided pots, and in most undivided pots, the root systems of neighboring plants could be separated before excavation of soil. For a small minority of pots (*n* < 10), the root systems of the neighboring plants were separated in the lab after water-filtering some of the soil surrounding the roots. A small amount of fine roots became disconnected from the larger root structures during this process, but in general, filtering whole blocks of soil should have effectively captured the majority of plant roots. Roots could be identified at the species level because of their attachment to the aboveground portion of the plants. Roots were then oven-dried (70°C for 48 h) before weighing to determine belowground biomass. After weighing, we re-hydrated each root sample with deionized water and determined root surface area using the program WinRhizo (Regent Instruments, Nepean, Ontario, Canada). Root samples were placed on a specialized scanner that, through the WinRhizo software, provided accurate estimates of many parameters including root surface area. We measured aboveground biomass near the height of the growing season (September 28) using nondestructive, allometric techniques (given above). This allometric equation was determined using individuals of 20 different locally collected genotypes of *S. altissima* and *S. gigantea* (Genung et al. 2012). In addition to the main stem, we also surveyed branches that were longer than 15 cm and treated these as additional “stems” for the purpose of the allometric equation. We found no difference in the relationship between height and biomass for *S. altissima* and *S. gigantea*; therefore, we use the same equation for both species. Similarly, we found no need to calculate a unique allometric equation for each genotype. We use the estimate of peak growing-season biomass as opposed to the final biomass because plants were harvested in early winter (10–12 December 2010) after leaves had senesced and dropped. Plants were harvested in early winter so that pollinator surveys could be carried out, although those data are not used in this study. Note that this means that aboveground biomass and belowground biomass were measured at different times and the results should be interpreted accordingly. In November, we also measured the number of new ramets (new stems at least 15 cm tall) produced by each plant. These data were incorporated into the allometric equation for aboveground biomass, and also analyzed as a response variable.

### Statistical methods

To determine whether excluding belowground interactions altered the effects of focal genotype and neighbor genotype on a range of plant traits, we used restricted maximum likelihood (REML) models. These models included the following terms: focal species, focal genotype, neighbor genotype, pot type (divided/undivided), the interaction of focal genotype and pot type, the interaction of neighbor genotype and pot type, and pot number (as a random effect). All nonrandom effects (except focal species) were nested within focal species. For this analysis we used only pots that included two species (i.e., monocultures were excluded), and because of this including neighbor species in the model would not provide any additional information (i.e., for a given focal species, the neighbor species was always the same). We were unable to include focal genotype by neighbor genotype interaction terms because labeling errors in our belowground subsample of pots prevented sufficient replication to run these models for belowground traits. Our response variables were aboveground biomass, belowground biomass, ramet production, and root surface area. Belowground biomass, aboveground biomass, and root surface area were transformed to meet assumptions of normality. An interaction between pot type and focal genotype would indicate that direct genetic effects are dependent upon the belowground subdivision treatment; similarly, an interaction between pot type and neighbor genotype would indicate the same for indirect genetic effects. To get a better idea of the drivers of these “focal genotype by divider” or “neighbor genotype by divider” interactions, we used post hoc contrasts (corrected for multiple testing using conservative reverse Bonferroni corrections) to determine if focal and neighbor genotype effects were significant in divided pots, undivided pots, both, or neither. We also used effect size measurements (Cohen's *d*) to determine whether the effects of focal genotype (or neighbor genotype) were more important, and to see if these effect size values were different, in divided and nondivided pots. We use the combined results of the post hoc contrasts and qualitative comparisons of effect size measurements to make inferences about the relative importance of focal genotype and neighbor genotype effects.

The analysis above included only pots containing two species, but we also wanted to test whether focal species or focal genotype identity interacted with the divider treatment in the monoculture pots. We used REML models with focal species, focal genotype, divider, and focal genotype by divider as model terms as pot number as a random effect. All nonrandom terms (except focal species) were nested within focal species. The remainder of this second analysis follows the same approach described above.

Because the plant traits we measured are likely to be correlated with each other, we calculated a correlation matrix for these traits. Additionally, we investigated whether root surface area or belowground biomass was a better predictor of aboveground biomass. We used the same model frameworks described above, except that either root surface area or belowground biomass was added as a predictor, and the only response variable was aboveground biomass.

## Results

### Does excluding belowground interactions alter the effects of focal genotype and neighbor genotype?

**Figure 2:**
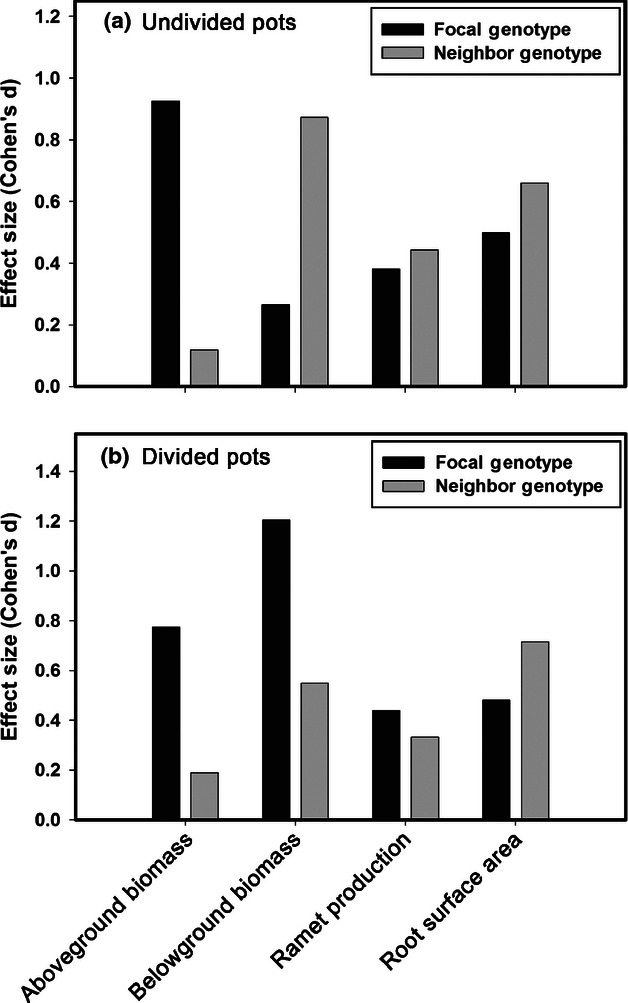
Belowground interactions shift the importance of genotype and neighbor genotype. The effect size by focal genotype and neighbor genotype varies depending on whether belowground interactions are allowed (a) or excluded (b). For most traits, trends were similar between divided and undivided pots. However, for belowground biomass in undivided pots, the effect size of neighbor genotype was qualitatively larger than the effect size of focal genotype. When calculating effect size, genotype and neighbor genotype were nested within species and neighbor species, respectively.

For two of the four traits, namely belowground biomass and ramet production, the effects of neighbor genotype identity depended on the exclusion of belowground interactions (Table 1). Post hoc tests indicated that genotypic variation in *S. altissima* neighbors affected the belowground biomass of *S. gigantea* focal plants in nondivided pots (*P* = 0.0273); in divided pots, we detected a nearly-significant effect (*P* = 0.0569). Genotype variation in *S. gigantea* neighbors did not affect belowground biomass in *S. altissima* focal plants in either belowground interactions treatment. Genotypic variation in *S. altissima* neighbors did not affect ramet production by *S. gigantea* focal plants in either belowground interactions treatment. Genotypic variation in *S. altissima* neighbors affected ramet production in *S. gigantea* focal plants in nondivided (*P* = 0.0034) but not divided (*P* = 0.2669) pots. It is worth noting that these traits are correlated with each other, and the results should be considered accordingly (Table 2). We also calculated effect sizes (Cohen's *d*) for focal genotype and neighbor genotype, and these were calculated separately for divided and nondivided pots. For the most part, the effect sizes for a given trait were similar in divided and nondivided pots, but belowground biomass showed a qualitative shift between the different pot types (Fig. 2). Together, these results point toward neighbor genotype having a stronger effect in nondivided than in divided pots.

**Table 1:** Excluding belowground interactions affects plant biomass allocation

Response	Factor	*N*	df	*F*	*P*
Aboveground	Focal Species	248	1	1.535	0.218
Biomass	***Focal Genotype (Sp.)***^1^		4	***19.448***	***<0.001***
	Neighbor Genotype (Sp.)		4	0.445	0.776
	Divider (Sp.)		2	0.872	0.420
	Genotype × Divider (Sp.)		4	0.957	0.432
	N. Genotype × Divider (Sp.)^2^		4	1.120	0.398
Belowground	Focal Species	61	1	0.083	0.776
Biomass	Focal Genotype (Sp.)		4	0.904	0.471
	Neighbor Genotype (Sp.)		4	0.214	0.929
	Divider (Sp.)		2	0.434	0.652
	Genotype × Divider (Sp.)		4	1.481	0.226
	***N. Genotype*** **×** ***Divider (Sp.)***		4	***3.383***	***0.018***
Ramet production	***Focal Species***	248	1	***48.392***	***<0.001***
	***Focal Genotype (Sp.)***		4	***5.281***	***<0.001***
	Neighbor Genotype (Sp.)		4	1.571	0.183
	Divider (Sp.)		2	0.565	0.211
	Genotype × Divider (Sp.)		4	0.915	0.456
	***N. Genotype*** **×** ***Divider (Sp.)***		4	***3.199***	***0.014***
Root surface area	Focal Species	56	1	0.790	0.382
	Focal Genotype (Sp.)		4	0.567	0.688
	Neighbor Genotype (Sp.)		4	0.761	0.558
	Divider (Sp.)		2	0.772	0.471
	Genotype × Divider (Sp.)		4	0.552	0.699
	N. Genotype × Divider (Sp.)		4	2.101	0.102

Results are shown for individuals of *Solidago altissima* and *Solidago gigantea* that were grown in divided and undivided pots. Two plants (one each of two species) were grown in each pot, and divided pots were separated belowground by a watertight, airtight barrier. All results come from REML models that also include pot number (experimental replicate) as a random effect. Bold, italicized values are significant at *α* = 0.05. The term “Focal Species” also incorporates, and is identical to, neighbor species identity as all pots include one *S. altissima* individual and one *S. gigantea* individual.

1 Sp., Species

2 N. Genotype, Neighbor Genotype.

**Table 2:** Correlations between measured plant traits

	Aboveground biomass	Belowground biomass	Ramet production	Root surface area
Aboveground biomass	–	***0.465***	***0.134***	***0.370***
Belowground biomass		–	***0.358***	***0.759***
Ramet production			–	***0.490***
Root surface area				–

We present data on four plant traits, and these traits are all correlated with each other. The strongest correlation is between belowground biomass and root surface area.

In two-species pots, species- and genotype-level variation in focal plants affected aboveground biomass and ramet production. Aboveground biomass was strongly determined by focal genotype identity, regardless of belowground interactions, suggesting that neighbors have little influence on a focal plant's total carbon allocation to aboveground structures. Production of new ramets was greater in *S. gigantea* than *S. altissima*, and genotypic variation for ramet production was present in *S. gigantea* (post hoc contrasts: *P* < 0.001) but not *S. altissima* (post hoc contrasts: *P* = 0.846). Given the strong effects of focal species and focal genotype on aboveground biomass and on ramet production, the lack of a similar effect on belowground biomass is surprising and suggests an important role for neighbor genotype. None of the factors were significant predictors of root surface area; this was surprising given that root surface area was tightly correlated with belowground biomass (Table 2). Considered alongside the results for “neighbor genotype by divider” interactions, these results show that focal genotype and neighbor genotype have different effects on different plant traits, and these effects can vary when belowground interactions are excluded.

### In genotype monocultures, do species and genotype identity affect plant traits?

Focusing on monocultures instead of species mixtures (and therefore removing the neighbor genotype terms and its interactions) did not qualitatively change our interpretation of focal species and focal genotype as drivers of belowground biomass and root surface area (Table 3). For these traits, we still did not detect an effect of focal species or focal genotype identity. Focal species joined focal genotype as a significant predictor of aboveground biomass, while focal species was the only significant predictor of ramet production. In no case did we see an effect of divider, or focal genotype by divider, in the monoculture pots.

**Table 3:** In genotype monocultures, focal genotype and focal species effects do not change when belowground interactions are excluded

Response	Factor	N	d.f.	F	p
Aboveground	***Focal Species***	166	1	***11.137***	***0.001***
Biomass	***Focal Genotype (Sp.)***^1^		4	***15.638***	***<0.001***
	Divider (Sp.)		2	0.388	0.680
	Genotype × Divider (Sp.)		4	0.517	0.724
Belowground	Focal Species	39	1	0.547	0.475
Biomass	Focal Genotype (Sp.)		4	1.422	0.288
	Divider (Sp.)		2	2.430	0.132
	Genotype × Divider (Sp.)		4	0.555	0.700
Ramet production	***Focal Species***	166	1	***37.751***	***<0.001***
	Focal Genotype (Sp.)		4	0.534	0.711
	Divider (Sp.)		2	0.282	0.755
	Genotype × Divider (Sp.)		4	0.373	0.827
Root surface area	Focal Species	36	1	1.296	0.275
	Focal Genotype (Sp.)		4	0.901	0.491
	Divider (Sp.)		2	0.841	0.453
	Genotype × Divider (Sp.)		4	0.246	0.908

Results are shown for individuals of *Solidago altissima* and *Solidago gigantea* that were grown in divided and undivided pots. Two plants (one each of two species) were grown in each pot, and divided pots were separated belowground by a watertight, airtight barrier. All results come from REML models that also include pot number (experimental replicate) as a random effect. Bold, italicized values are significant at *α* = 0.05.

1 Sp, Species.

### What better predicts aboveground biomass – root surface area or belowground biomass?

Belowground biomass and root surface area can both be indications of a plant's ability to acquire belowground nutrients, so we examined which of these traits was a better predictor of aboveground biomass. We found that belowground biomass was a significant predictor of aboveground biomass in monoculture pots (*F*_(1, 39)_ = 8.683, *P* = 0.007) and in species mixture pots (*F*_(1, 61)_ = 9.258, *P* = 0.004). In contrast, root surface area was a marginally significant predictor of aboveground biomass in monoculture pots (*F*_(1, 36)_ = 3.355, *P* = 0.080) and in species mixture pots (*F*_(1, 56)_ = 3.193, *P* = 0.084).

## Discussion

Overall, we found that the presence of belowground interactions altered how IIGEs (i.e., neighbor genotype effects) from neighboring plants affected belowground biomass in focal plants, and that the effect size of genotype and neighbor genotype (Fig. 2) varied across plant traits and environmental conditions. These results help inform how the relative importance of direct (focal genotype) and indirect (neighbor genotype) genetic effects may vary, depending on the trait in question and how the neighboring plants are interacting. At a broader scale, the relatively large roles of focal genotype and neighbor genotype help inform the effort to identity the importance of genotypic variation relative to other ecological factors (e.g., Bailey et al. 2009).

It is well known that an individual's phenotype is the result of interacting genetic and environmental influences, and in this study we found that genotypic variation and IIGEs were contingent on an experimental manipulation of the “environment” – specifically, whether belowground interactions were allowed or excluded. This environmental manipulation shifted the effect size of focal genotype and neighbor genotype (Fig. 2), but only for the belowground biomass trait. One explanation for this pattern is that focal genotype effects were partially counteracted by the effects of neighbor genotype in undivided pots. We found that none of our factors predicted root surface area, and that belowground biomass performed better than root surface area as a predictor of aboveground biomass. While root surface area can provide more insight into belowground competition than belowground biomass (Caldwell et al. 1991; Casper and Jackson 1997), there are scenarios under which the relationship between root surface area and competition break down. Plants can temporally or spatially partition the way they acquire nutrients such that nutrient depletion zones do not overlap (Mooney et al. 1986; Fernandez and Caldwell 1975), the location within the soil where roots are deployed (i.e., areas of high nutrient density or low nutrient density) can override the effects of root surface area, or root competition can occur between the roots of the same plant (Casper and Jackson 1997). We did not detect any evidence of neighboring plants facilitating each other's growth by partitioning the way they acquire resources, because neither above- nor belowground biomass were affected by the main effect of pot division (Table 1). The context-dependent (i.e., dependent on belowground interactions) effects of neighbor genotype indicate that neighbor genotype effects vary depending on whether plants are allowed to interact belowground.

Similar to the results of a previous study (Genung et al. 2012), we found that IIGEs played a role in determining belowground biomass. When plants were allowed to interact belowground, IIGEs had a larger effect on belowground biomass than did genotype (Fig. 2). This pattern is likely driven by intense belowground competition in a non light limited environment (Wilson 1988; Wilson and Tilman 1993). This observation extends the results of our previous work (Genung et al. 2012) by explicitly supporting the hypothesis that neighbor genotype effects are, overall, stronger when plants are allowed to interact belowground. Additionally, because the effect size of neighbor genotype was larger than focal genotype for belowground biomass, this result also suggests that, at least for belowground traits in *Solidago*, focal plant genotypic variation is more related to exerting IIGEs on neighbors than to biomass production in the focal plant. One possible mechanism for this pattern involves allelopathy, through which plants exude chemicals that can positively or negatively affect interacting organisms (see Schenk 2006 for review). *Solidago* is known to produce allelopathic chemicals, specifically polyacetylenes and diterpenes (Hegnauer 1977). Allelopathy allows *Solidago* to negatively affect neighboring species, especially those without a shared coevolutionary history, for example, when invading European ecosystems (Abhilasha et al. 2008). Although we did not test for the potential effects of allelopathy, the strong effects of IIGEs on belowground biomass production of focal plants warrant further investigation.

Our results provide a novel perspective on the importance of direct versus indirect genetic effects in plant-neighbor interactions by showing that, in *Solidago*, a focal plant's belowground biomass phenotype can be strongly determined by IIGEs from its neighbor. This observation has important implications for coevolutionary processes acting on the interacting plants (Dawkins 1982; Moore et al. 1997; Shuster et al. 2006; Wade 2007). Wade (2007) wrote that community genetics may change the amount of information that can be attached to genes, and our results suggest that genes may have predictable effects not only on the organism in which they are expressed but also on neighboring individuals. Furthermore, neighbor genotype effects suggest that the fitness consequences of a given trait for a focal plant should be correlated with the fitness consequences for neighboring plants, meaning that any change in environmental conditions could indirectly affect a plant's fitness by altering traits in its neighbors. Our results suggest that plant competition studies at the genotype level should measure both above- and belowground biomass, especially if they are interested in correctly understanding the influence of neighbors in nutrient limited environments (Genung et al. 2012). For example, neighboring plants may have large effects on each other's belowground biomass, which may not be apparent from patterns of aboveground biomass (Fig. 2) but nonetheless can affect the fitness of the interacting plants. While it is becoming better known that IIGEs have important ecological and coevolutionary consequences (Shuster et al. 2006; Whitham et al. 2011), our results provide a new case study that shows that belowground interactions can be a mechanism for IIGEs. Interesting possibilities for future work involve determining whether, for belowground biomass traits, evolutionary causes have driven IIGEs to be strong relative to focal genotype effects, and developing better mechanistic understandings of how these belowground IIGEs occur.

## References

[b1] Abhilasha D, Quintana N, Vivanco J, Joshi J (2008). Do allelopathic compounds in invasive *Solidago canadensis* s.l. restrain the native European flora?. J. Ecol.

[b2] Abrahamson WG, Weis AE (1997). Evolutionary ecology across three trophic levels: goldenrods, gallmakers, and natural enemies.

[b3] Abrahamson WG, Dobley KB, Houseknecht HR, Pecone CA (2005). Ecological divergence among five co-occuring species of old-field goldenrods. Plant Ecol.

[b4] Bailey JK, Wooley SC, Lindroth RL, Whitham TG (2006). Importance of species interactions to community heritability: a genetic basis to trophic-level interactions. Ecol. Lett.

[b5] Bailey JK, Schweitzer JA, Ubeda F, Koricheva J, LeRoy CJ, Madritch MD (2009). From genes to ecosystems: a synthesis of the effects of plant genetic factors across levels of organization. Philos. Trans. R. Soc. Lond. B Biol. Sci.

[b6] Bossdorf O, Shuja Z, Banta JA (2009). Genotype and maternal environment affect belowground interactions between *Arabidopsis thaliana* and its competitors. Oikos.

[b7] Caldwell MM, Manwaring JH, Jackson RB (1991). Exploitation of phosphate from fertile soil microbes by 3 Great Basin perennials when in competition. Funct. Ecol.

[b8] Casper BB, Jackson RB (1997). Plant competition underground. Annu. Rev. Ecol. Syst.

[b9] Collins A, Hart EM, Molovsky J (2010). Differential response to frequency-dependent interactions: an experimental test using genotypes of an invasive grass. Oecologia.

[b10] Crutsinger GM, Collins MD, Fordyce JA, Gompert Z, Nice CC, Sanders NJ (2006). Plant genotypic diversity predicts community structure and governs an ecosystem process. Science.

[b11] Crutsinger GM, Sanders NJ, Classen AT (2009). Comparing intra- and inter-specific effects on litter decomposition in an old-field ecosystem. Basic Appl. Ecol.

[b12] Dawkins R (1982). The extended phenotype: the gene as the unit of selection.

[b13] Fernandez OA, Caldwell MM (1975). Phenology and dynamics of root growth of three cool semi-desert shrubs under field conditions. J. Ecol.

[b14] Fridley JD, Grime JP, Bilton M (2007). Genetic identity of interspecific neighbours mediates plant responses to competition and environmental variation in a species-rich grassland. J. Ecol.

[b15] Genung MA, Bailey JK, Schweitzer JA (2012). Welcome to the neighbourhood: interspecific genotype by genotype interactions in *Solidago* influence above- and belowground biomass and associated communities. Ecol. Lett.

[b16] Hegnauer R, Heywood VH, Harborne JB, Turner BL (1977). The chemistry of compositae. The biology and chemistry of the compositae.

[b18] Johnson MTJ, Agrawal AA (2005). Plant genotype and environment interact to shape a diverse arthropod community on evening primrose (*Oenothera biennis*. Ecology.

[b19] Johnson MTJ, Lajeunesse MJ, Agrawal AA (2006). Additive and interactive effects of plant genotypic diversity on arthropod communities and plant fitness. Ecol. Lett.

[b20] Maddox GD, Root RB (1987). Resistance to 16 diverse species of herbivorous insects within a population of goldenrod, *Solidago altissima*: genetic variation and heritability. Oecologia.

[b21] Madritch M, Donaldson JR, Lindroth RL (2006). Genetic identity of *Populus tremuloides* litter influences decomposition and nutrient release in a mixed forest stand. Ecosystems.

[b22] Mooney KA, Agrawal AA (2008). Plant genotype shapes ant-aphid interactions: implications for community structure and indirect plant defense. Am. Nat.

[b23] Mooney HA, Hobbs RJ, Gorham J, Williams K (1986). Biomass accumulation and resource utilization in cooccurring grassland annuals. Oecologia.

[b24] Moore AJ, Brodie ED, Wolf JB (1997). Interacting phenotypes and the evolutionary process. 1. Direct and indirect genetic effects of social interactions. Evolution.

[b25] Pregitzer CC, Bailey JK, Hart SC, Schweitzer JA (2010). Soils as agents of selection: feedbacks between plants and soils alter seedling survival and performance. Evol. Ecol.

[b26] Schenk HJ (2006). Root competition: beyond resource depletion. J. Ecol.

[b27] Schmid B, Bazzaz FA (1992). Growth responses of rhizomatous plants to fertilizer application and interference. Oikos.

[b28] Schweitzer JA, Bailey JK, Rehill BJ, Martinsen GD, Hart SC, Lindroth RL (2004). Genetically based trait in a dominant tree affects ecosystem processes. Ecol. Lett.

[b29] Schweitzer JA, Bailey JK, Fischer DG, Leroy CJ, Lonsdorf EV, Whitham TG (2008). Plant-soil-microorganism interactions: heritable relationship between plant genotype and associated soil microorganisms. Ecology.

[b30] Shuster SM, Lonsdorf EV, Wimp GM, Bailey JK, Whitham TG (2006). Community heritability measures the evolutionary consequences of indirect genetic effects on community structure. Evolution.

[b31] Wade MJ (2007). The co-evolutionary genetics of ecological communities. Nat. Rev. Genet.

[b32] Whitham TG, Bailey JK, Schweitzer JA, Shuster SM, Bangert RK, LeRoy CJ (2006). A framework for community and ecosystem genetics: from genes to ecosystems. Nat. Rev. Genet.

[b33] Whitham TG, Gehring CA, Lamit LJ, Wojtowicz T, Evans LM, Keith AR (2011). Community specificity: life and afterlife effects of genes. Trends Plant Sci.

[b34] Wilson JB (1988). Shoot competition and root competition. J. Appl. Ecol.

[b35] Wilson SD, Tilman D (1993). Plant competition and resource availability in response to disturbance and fertilization. Ecology.

